# Withdrawn

**DOI:** 10.1002/fsn3.2147

**Published:** 2021-01-23

Withdrawal: Ahmad N, Hussain M, Sameen A, et al. Extraction and enrichment of maize bran cell wall in bread and its textural, and microbial assessment. Food Sci Nutr. 2021;00:1‐8. https://doi.org/10.1002/fsn3.2147 The above article from Food Science & Nutrition, published online on 23 January 2021 in Wiley Online Library as Early View (https://onlinelibrary.wiley.com/doi/epdf/10.1002/fsn3.2147), has been withdrawn by agreement among the authors, the Editor‐in‐Chief, and Wiley Periodicals LLC. The withdrawal has been agreed because the article has been submitted and approved for publication by Emad Karrar without consent by several co‐authors.

